# Social inequalities shape diet composition among urban Colombians: the Colombian Nutritional Profiles cross-sectional study

**DOI:** 10.1017/S1368980021004778

**Published:** 2021-12-10

**Authors:** Pedro J Quiroga-Padilla, Paula V Gaete, Luz D Nieves-Barreto, Angélica Montaño, Eddy C Betancourt, Carlos O Mendivil

**Affiliations:** 1 Universidad de los Andes, School of Medicine, Carrera 7 No 116-05, Of 413, Bogotá 110111, Colombia; 2 Team Foods Colombia, Bogotá, Colombia; 3 Fundación Santa Fe de Bogotá, Section of Endocrinology, Bogotá, Colombia

**Keywords:** Diet, Socio-economic position, Urban, Inequality, Risk factors, Education

## Abstract

**Objective:**

To explore the influence of socio-economic position (SEP) on habitual dietary intake in Colombian cities.

**Design:**

We conducted a cross-sectional, population-based study in five Colombian cities. Dietary intake was assessed with a 157-item semi-quantitative FFQ previously developed for the Colombian population. Nutrient analysis was performed using national and international food composition tables. SEP was assessed with two indicators: a government-defined, asset-based, household-level index called socio-economic stratum (SES) and, among adults, highest educational level attained.

**Setting:**

The five main urban centers of Colombia: Bogotá, Medellin, Barranquilla, Cali and Bucaramanga.

**Participants:**

Probabilistic, multi-stage sample of 1865 participants (*n* 1491 for analyses on education).

**Results:**

For both sexes, increasing SES was associated with a lower consumption of energy (*P*-trend <0·001 in both sexes), carbohydrates (*P*-trend <0·001 in both sexes), Na (*P*-trend = 0·005 in males, <0·001 in females), SFA (*P*-trend <0·001 in both sexes) and among females, cholesterol (*P*-trend = 0·002). More educated men consumed significantly less energy and carbohydrates (*P*-trend = 0·036 and <0·001, respectively). Among men, intake of trans fats increased monotonically with educational level, being 21 % higher among college graduates relative to those with only elementary education (*P*-trend = 0·023). Among women, higher educational level was associated with higher MUFA intake (*P*-trend = 0·027).

**Conclusions:**

SES and educational level are strong correlates of the usual diet of urban Colombians. Economically deprived and less educated segments of society display dietary habits that make them vulnerable to chronic diseases and should be the primary target of public health nutrition policies.

Social inequalities are key determinants of health and disease across different populations^(1,2)^. Health indicators like life expectancy or infant mortality have a stepwise association with socio-economic position (SEP), with health improving incrementally as social position rises^(2)^. Evidence from a multinational study of older individuals undertaken in five Latin American countries, India and China, showed that multiple indicators of SEP including education, occupational attainment, assets and pension receipt were inversely associated with total mortality over a 5-year period^(3)^.

Diet may constitute a crucial link between social inequalities and disease. Obesity^(4)^, diabetes, IHD, stroke and cancer are known to be associated with both SEP and diet quality^(5,6)^. Additionally, and despite methodological differences, a large number of studies show a significant influence of social status over energy, macro- and micronutrient intake^(7)^. A systematic review of cross-sectional studies from low- and middle-income countries found that higher SEP was associated with a larger intake of energy, protein and all fat subtypes, and with lower intakes of carbohydrates and fibre^(8)^.

Although several Latin American countries^(9–11)^ conduct nationally representative nutritional surveys, the association between socio-economic variables and dietary composition has been explored mostly in Brazil and Mexico. The first Brazilian National Dietary Survey (2008–2009) included 13 569 households and found that energy intake was positively and independently associated with income and years of education for both sexes^(10)^. In the last National Health and Nutrition Survey of Mexico (ENSANUT 2012), which included 10 886 households^(9)^, higher SEP was associated with increased energy intake but reduced carbohydrate consumption for both sexes. A prior small, single-city study in Bucaramanga, Colombia, examined dietary intake using a 7-d food diary and encountered a positive correlation of SEP with protein and total fat intake and a negative correlation with carbohydrate intake^(12)^.

Colombia and other middle-income Latin American countries are currently undergoing an epidemiological and nutritional transition, in which non-communicable diseases represent an ever-increasing share of the disease and mortality burden, while acute and infectious diseases have not been completely eradicated^(13)^. Despite its developing country status, non-communicable causes are by far the most significant source of morbidity and mortality in Colombia. This is well illustrated by the fact that CVD, cerebrovascular disease, diabetes and cancer accounted for 68 % of the total deaths in 2015^(14)^. Disorders of energy balance and metabolism have a deep influence on the risk of these conditions, both directly and through their effect on mediating risk factors like BMI, blood pressure, blood lipids and diabetes. The prevalence of overweight and obesity from the five main cities of Colombia in 2018 was 57·5 % in adults (36·2 % overweight, 21·3 % obesity)^(15)^ and 31·8 % in children and adolescents (23·0 % overweight, 8·8 % obesity). These extremely worrisome figures place Colombia close to countries with a massive epidemic of overweight and obesity like Mexico and the USA. Given that dietary behaviour is a major determinant of energy balance and the risk of developing non-communicable diseases, knowledge of dietary intake and its major correlates is of essential importance for the country. Among these correlates, factors related to SEP have a preeminent relevance.

In spite of the existing evidence about the association between diet composition and indicators of social standing, this relationship and its magnitude have not been extensively studied in Latin America. In order to design and implement successful public health policies, it is imperative to assess the relative contribution of factors like income and education to the nutritional profile of different segments of the population. With this motivation, the present study aimed to dissect the influence of two relevant indicators of SEP: socio-economic stratum (SES) and educational level, on habitual dietary intake in five Colombian cities and to explore whether such influences differ between men and women.

## Methods

### Study area

Colombia is a Latin-American country, located at the northwestern tip of South America. In 2018, Colombia had an estimated population of 48 million, 78 % of whom lived in urban areas^(16)^. Colombia has a Human Development Index of 0·767, ranking eighty-three out of 189 countries^(17)^. Similar to other Latin American countries, Colombia is characterised by a marked difference in poverty levels and economic development between rural and urban locations. The prevalence of multidimensional poverty is almost 3 times larger in rural than urban areas, mostly due to differences in access to public services and literacy levels^(18)^. The five cities included in this study (Bogota, Medellin, Barranquilla, Cali and Bucaramanga) comprise approximately 30 % of the Colombian population and 38 % of the Colombian urban population^(16)^.

### Sampling and data collection

COPEN (Estudio Colombiano de Perfiles Nutricionales – Colombian Study of Nutritional Profiles) was a population-based, cross-sectional, multi-stage sampling survey designed to represent five cities, one from each of Colombia’s major regions. The sampling frame was obtained from the last (2005) census of the Colombian population^(19)^, cartography was obtained from the national geostatistical frame developed by the Colombian National Department of Statistics and data on SES came from the National Superintendence of Public Services. In the first stage of sampling, we selected cartographic sectors, within sectors we selected blocks (on average eight per cartographic sector), within blocks we selected households and within households we selected individual participants. All individuals over the age of 2 were listed and a person was randomly selected. In the case of participants under the age of 13, information was provided by the adult responsible for the participant. The sample was stratified by city, sex, age group and SES of the household.

All data were collected between June and November 2018. Information was captured using a tablet device containing digital forms with proper validation rules, developed for the study. All staff in charge of data collection was extensively trained by the study Principal Investigator. A random 10 % of participants were re-contacted by phone to double-check the accuracy of the information provided on the date of birth, sex, city of residence, marital status, job status, educational level and date of initial contact. With this design and including the design effect, the study sample yielded an overall sampling error of 2·2 % for the prevalence of overweight or obesity in the target population, which was a central objective of the COPEN study. The sampling errors for each city were respectively: Bogota 4·0 %, Medellin 5·0 %, Cali 5·0 %, Barranquilla 5·6 % and Bucaramanga 6·8 %.

### Participants

Participants were individuals between the ages of 2 and 75, residing in one of the five cities mentioned above. We excluded foreigners living in Colombia, individuals in haemodialysis or peritoneal dialysis therapy and persons with disabilities that precluded a reliable fulfilment of the study questionnaire.

### Socio-demographic and anthropometric variables

We collected information on sex, date of birth and household SES (in all participants), and marital status, individual educational level and employment status (in participants aged 18 or older), using a standardised questionnaire. SES is classified in Colombia by the Statistics Department DANE in six strata according to characteristics of the residence (with stratum 1 being the lowest and stratum 6 being the highest)^(20)^. Residential dwellings are classified according to their physical characteristics and environment. The methodology for this classification creates homogeneous strata taking as input information about land use, public utilities, access routes, topography, land valuation and property characteristics. Residential dwellings are classified in the predominant stratum of the sub-zone, as long as their characteristics do not differ ostensibly from the predominant conditions in the group. Otherwise, they are considered outliers and their stratum is assessed based on their particular characteristics. This information is very well established, updated and freely accessible for all the country^(21)^. It also has a significant correlation with household income. A single score is created, converting and weighing each variable with the Savage score method. Living places are classified in six strata, according to a cluster analysis (with stratum 1 being the lowest and stratum 6 being the highest). Given that socio-demographic, income and human development indicators are more similar for individuals living in strata 4–6 than among the other strata^(21)^, we analysed SES in three groups, corresponding to strata 1–2 (low SES), 3 (medium SES) and 4–6 (high SES). Participants were asked to report what was the highest educational cycle they had completed: pre-school, primary school, secondary school (lasting 6 years, there is no equivalent of high school in Colombia), technical degree, college degree or post-graduate degree. For the effects of analyses, and in order to make findings more comparable with international standards, the variable educational level was operationalised in three categories as: elementary or lower, secondary or technical degree, and college or higher. Only participants aged 18 or older were asked about their educational level, as many underage individuals may still be completing their education. Hence, all analyses involving educational level include only adult participants and have a different sample size. Height was measured using a portable stadiometer supported on a firm surface. Weight was measured employing a solar digital scale with 100 g sensitivity and 200 kg capacity. For the characterisation of the study sample, we analysed BMI in participants under 18 years of age as the *Z*-score of the sex-specific BMI-for-age curves.

### Food frequency questionnaire

Dietary intake was assessed using a semi-quantitative FFQ with a 157-item food list, plus frequency of intake and number of standard portions consumed (with reference portion size written next to this field). This FFQ had been previously developed and piloted in the Colombian population^(22)^. The Colombian National Nutritional Situation Survey (*Encuesta Nacional de Situación Nutricional*) in its 2005 version performed a 24-h dietary recall (this was omitted from later versions of *Encuesta Nacional de Situación Nutricional*). This dietary recall was applied in a randomly selected day of the week to 39 413 non-pregnant male and female participants aged 2–64 years (data were provided by the responsible adult in the case of persons aged less than 13). A second 24-h dietary recall was performed in a random subsample of 3534 participants, in a second, non-consecutive day. Employing the compiled results from this dietary recall, foods were ranked from most to least frequently consumed^(23)^. From the 372 foods listed, 142 were consumed by at least 30 % of the studied population. To this list, three typical regional foods from each of the five regions studied were added, resulting in 157 food items in the FFQ. Portion sizes were calculated according to the coding of weights and measurements in *Encuesta Nacional de Situación Nutricional* 2005; the unit of measure most frequently reported for each food was used as reference portion size^(22)^. The average frequency of intake for each food item over the last year was registered as one of nine categories: never, 1–3 times/month, once a week, 2–4 times a week, 5–6 times a week, once a day, 2–3 times a day, 4–6 times a day or more than 6 times a day. A trained staff member administered the FFQ and registered all the information.

Estimation of daily nutrient intake was done as previously described^(24)^. First, a weighing factor was used to convert each frequency of intake to number of portions consumed in a day. Then a factor was used to convert the number of portions a day to 100-g units. Subsequently, an edible fraction factor was applied. Composition data were obtained from the Colombian Institute of Family Welfare (Instituto Colombiano de Bienestar Familiar) reference tables^(25)^. For foods not in Instituto Colombiano de Bienestar Familiar tables, composition was extracted from the Central America and Panama Nutrition Institute (Instituto de Nutrición de Centro América y Panamá) tables^(26)^ or the US Department of Agriculture food composition database (FoodData Central)^(27)^. For foods not represented in any of these sources, information from the manufacturer was employed. For analyses purposes, we expressed total energy consumption in kJ/kg per d (kcal/kg per d). The consumption of carbohydrates, protein, lipids, SFA, PUFA and MUFA was expressed in g/kg per d, in order to compare diet composition removing the effect of body size. We also analysed differences in the percentage daily kilojoules (kcal) coming from each macronutrient across categories of SES and educational level. Nutrients providing no or negligible energy were analysed in weight units, namely mg/d for cholesterol, g/d for trans fats, g/d for fibre and mg/d for Na.

### Data analysis

All estimations were projected to the target study population using city, sex, age group and SES-specific expansion factors according to the study multi-stage sampling design. The mean daily kilojoules (kcal), macronutrients and lipid subtypes (per kg body weight), fibre, cholesterol and Na were compared across categories of categorical predictors using a one-way linear model (ANOVA). We focused on these nutrients because of their proven association with the risk of chronic diseases. When global ANOVA was significant, post-hoc pairwise comparisons were done against a reference category (the lowest) using Dunnett’s method. All analyses were two-tailed and carried out at a 5 % significance level. All analyses were performed in SPSS for Windows, v.21.

## Results

The study sample included 1865 participants (914 male and 951 female), most of whom were residents of Bogota (32·2 %), followed by Cali (21·1 %), Medellin (19·8 %), Barranquilla (15·7 %) and Bucaramanga (11·2 %) (Table 1). Mean BMI was 28·0 kg/m^2^ in female and 25·7 kg/m^2^ in male adult participants. Only a quarter of participants lived in high SES, and most (44·7 %) lived in low SES households. The most prevalent educational level was secondary or technical, and only 20·3 % had a college or higher degree. The age distribution of the sample resembled the Colombian population pyramid^(16)^.

**Table 1:** Characteristics of the study participants

	Male		Female		Total	
	*n*	%	*n*	%	*n*	%
*n*	914	49·0 %	951	51·0 %	1865	100 %
Age group, years						
2–11	143	15·6 %	83	8·7 %	226	12·1 %
12–17	88	9·6 %	60	6·3 %	148	7·9 %
18–39	287	31·4 %	325	34·2 %	612	32·8 %
40–59	204	22·3 %	263	27·6 %	467	25·0 %
60–75	192	21·0 %	220	23·1 %	412	22·1 %
Socio-economic stratum						
Low	411	45·0 %	423	44·5 %	834	44·7 %
Medium	273	29·9 %	281	29·5 %	554	29·7 %
High	230	25·2 %	247	26·0 %	477	25·6 %
City						
Barranquilla	145	15·9 %	148	15·6 %	293	15·7 %
Bogota	295	32·3 %	305	32·1 %	600	32·2 %
Bucaramanga	103	11·3 %	106	11·1 %	209	11·2 %
Cali	192	21·0 %	202	21·2 %	394	21·1 %
Medellin	179	19·6 %	190	20·0 %	369	19·8 %
Educational level*						
Elementary or lower	137	20·1 %	177	21·9 %	314	21·1 %
Secondary or technical degree	402	58·9 %	472	58·3 %	874	58·6 %
College or higher	143	21·0 %	160	19·8 %	303	20·3 %
Weight among adults (kg)	74·5	15·4	67·3	13·3	70·6	14·8
Weight-for-age *Z*-score among minors						
Mean	−0·18		0·02		−0·10	
SD	1·46		0·91		1·25	
Height among adults (cm)						
Mean	169·7		155·7		162·1	
SD	7·2		8·6		10·6	
Height-for-age *Z*-score among minors						
Mean	−0·46		−0·38		−0·43	
SD	1·15		1·09		1·13	
BMI among adults (kg/m^2^)						
Mean	25·8		28·0		27·0	
SD	4·6		8·9		7·3	
*Z*-score of BMI-for-age among minors						
Mean	0·39		0·58		0·46	
SD	1·40		2·5		1·88	

* Educational level only for participants aged 18 or older (*n* 1491).

Data are expressed as *n* (%) or mean (SD).

Female sex was associated with a lower intake of energy, all macronutrients (even on a per kg basis) and Na (*P* < 0·001 for all comparisons). In both sexes, there was a significant decreasing trend in the total intake of energy with increasing levels of SES. For males, the values went from 337 kJ/kg per d (80·5 kcal/kg per d) in low SES to 261 kJ/kg per d (62·3 kcal/kg per d) in high SES (*P*-trend < 0·001), while for females they went from 301 kJ/kg per d (72 kcal/kg per d) in low SES to 233 kJ/kg per d (55·8 kcal/kg per d) in high SES (*P*-trend < 0·001). The largest difference, however, was observed between participants in medium SES and those in high SES (Table 2). A similar result was observed for carbohydrates, which went from 10·0 g/kg per d in low SES to 7·6 g/kg per d in high SES among males (*P*-trend < 0·001), and from 8·9 g/kg per d to 6·4 g/kg per d among females (*P*-trend <0·001). The intake of all fat subtypes decreased with SES in both sexes, except for MUFA among females.

**Table 2:** Estimated intake of energy, macronutrients, trans fats, Na, fibre and cholesterol, by sex and socio-economic stratum

Males (*n* 914)									
	Low SES (*n* 411)		Medium SES (*n* 273)			High SES (*n* 230)			*P*-trend
	Mean	95 % CI	Mean	95 % CI	*P v* low	Mean	95 % CI	*P v* low	
Energy (kcal/kg per d)	80·5	73·4, 87·6	75·8	68·6, 83	0·008	62·3	58·3, 66·3	<0·001	<0·001
Energy (kJ/kg per day)	337	307, 366	317	287, 347	0·008	261	244, 277	<0·001	<0·001
Protein (g/kg per d)	2·81	2·6, 3·1	2·65	2·4, 2·9	0·013	2·29	2·1, 2·4	<0·001	0·03
Carbohydrates (g/kg per d)	10·0	9·1, 10·9	9·5	8·6, 10·4	0·015	7·6	7·0, 8·2	<0·001	<0·001
Lipids (g/kg per d)	3·16	2·8, 3·5	2·94	2·6, 3·2	0·006	2·41	2·2, 2·6	<0·001	<0·001
SFA (g/kg per d)	1·05	0·93, 1·17	0·97	0·87, 1·07	0·009	0·81	0·75, 0·87	<0·001	<0·001
MUFA (g/kg per d)	1·24	1·11, 1·37	1·19	1·07, 1·31	0·051	1·05	0·98, 1·12	<0·001	<0·001
PUFA (g/kg per d)	0·75	0·67, 0·83	0·66	0·58, 0·74	0·006	0·53	0·49, 0·57	<0·001	<0·001
Cholesterol (mg/d)	773	723, 823	723	666, 780	–	663	605, 721	–	0·16
Trans fats (g/d)	2·17	2·0, 2·3	2·26	2·1, 2·5	–	2·49	2·3, 2·7	–	0·19
Fibre (g/d)	37·0	35·5, 38·5	36·5	34·8, 38·2	–	36·3	34·3, 38·3	–	0·79
Na (mg/d)	5898	5647, 6149	5391	5129, 5653	0·004	5315	4987, 5643	0·025	0·005
Females (*n* 951)									
	Low SES (*n* 423)		Medium SES (*n* 281)			High SES (*n* 247)			
Energy (kcal/kg per d)	72·0	66·8, 77·2	73·8	67·0, 80·6	0·96	55·8	52·5, 59·1	<0·001	<0·001
Energy (kJ/kg per d)	301	279, 323	309	280, 337	0·96	233	220, 247	<0·001	<0·001
Protein (g/kg per d)	2·51	2·3, 2·7	2·55	2·3, 2·8	0·99	2·02	1·9, 2·1	<0·001	0·001
Carbohydrates (g/kg per d)	8·9	8·2, 9·6	9·4	8·5, 10·3	0·76	6·4	6·0, 6·8	<0·001	<0·001
Lipids (g/kg per d)	2·86	2·6, 3·1	2·85	2·6, 3·1	0·97	2·43	2·3, 2·6	0·001	0·001
SFA (g/kg per d)	0·93	0·85, 1·01	0·91	0·82, 1·00	0·81	0·84	0·78, 0·90	0·003	0·002
MUFA (g/kg per d)	1·13	1·04, 1·22	1·15	1·04, 1·26	–	1·04	0·98, 1·10	–	0·14
PUFA (g/kg per d)	0·69	0·63, 0·75	0·70	0·63, 0·77	0·95	0·52	0·48, 0·56	<0·001	0·001
Cholesterol (mg/d)	642	597, 687	641	590, 692	0·37	560	522, 598	0·003	0·002
Trans fats (g/d)	2·0	1·8, 2·2	2·02	1·8, 2·2	–	2·2	2·0, 2·4	–	0·63
Fibre (g/d)	33·1	31·6, 34·6	32·4	30·8, 34·0	–	33·8	32·1, 35·5	–	0·42
Na (mg/d)	5132	4913, 5351	5058	4797, 5319	0·84	4786	4546, 5026	<0·001	<0·001

The *P*-value for the univariate comparison against the lowest educational level (Dunnett’s test) is shown only when global ANOVA was significant.

Meanwhile, dietary patterns by educational level did not display such a clear tendency. Energy intake decreased with increasing education among men, going from 219 kJ/kg per d (52·4 kcal/kg per d) among those with elementary education to 194 kJ/kg per d (46·3 kcal/kg per d) among college graduates (*P*-trend = 0·036) (Table 3). More educated men also consumed less carbohydrates (6·71 g/kg per d in the lowest category *v* 5·25 g/kg per d in the highest, *P*-trend < 0·001). Women with secondary or college education displayed a higher intake of MUFA (0·77 g/kg per d in the lowest category *v* 0·89 g/kg per d in the highest, *P*-trend = 0·027).

**Table 3:** Estimated intake of energy, macronutrients, trans fats, Na, fibre and cholesterol, by sex and educational level. These analyses included only participants aged 18 or older

Men (*n* 682)									
	Elementary or lower (n 137)		Secondary or technical degree (*n* 402)			Professional or higher (n 143)			*P*-trend
	Mean	95 % CI	Mean	95 % CI	*P v* elementary	Mean	95 % CI	*P v* elementary	
Energy (kcal/kg per d)	52·4	48·0, 56·8	54·2	51·6, 56·7	0·67	46·3	42·7, 49·8	0·07	0·036
Energy (kJ/kg per d)	219	201, 238	227	216, 237	0·67	194	179, 208	0·07	0·036
Protein (g/kg per d)	1·81	1·6, 2·0	1·93	1·8, 2·0	–	1·74	1·6, 1·9	–	0·49
Carbohydrates (g/kg per d)	6·71	6·1, 7·3	6·64	6·3, 7·0	0·96	5·25	4·8, 5·7	<0·001	<0·001
Lipids (g/kg per d)	1·92	1·7, 2·1	2·09	2·0, 2·2	–	1·91	1·7, 2·1	–	0·95
SFA (g/kg per d)	0·60	0·54, 0·67	0·68	0·64, 0·72	–	0·61	0·55, 0·67	–	0·94
MUFA (g/kg per d)	0·76	0·68, 0·84	0·87	0·82, 0·91	0·04	0·85	0·77, 0·93	0·17	0·11
PUFA (g/kg per d)	0·45	0·39, 0·50	0·49	0·46, 0·52	–	0·42	0·37, 0·47	–	0·46
Cholesterol (mg/d)	659	581, 737	718	668, 768	–	679	607, 750	–	0·75
Trans fats (g/d)	1·86	1·6, 2·1	2·15	2·0, 2·3	0·082	2·26	2·0, 2·5	0·04	0·023
Fibre (g/d)	36·0	33·3, 38·7	36·2	34·7, 37·7	–	33·8	31·3, 36·3	–	0·22
Na (mg/d)	5061	4631, 5491	5270	5028, 5512	–	4530	4192, 4867	–	0·056
Women (*n* 809)									
	Elementary or lower (*n* 177)		Secondary or technical degree (*n* 472)			Professional or higher (*n* 160)			
Energy (kcal/kg per d)	47·8	43·6, 52·1	53·0	50·7, 55·3	0·034	47·6	44·3, 50·8	0·99	0·98
Energy (kJ/kg per d)	200	182, 218	222	212, 231	0·034	199	185, 212	0·99	0·98
Protein (g/kg per d)	1·62	1·5, 1·8	1·84	1·8, 1·9	0·01	1·74	1·6, 1·9	0·37	0·19
Carbohydrates (g/kg per d)	5·87	5·3, 6·4	6·30	6·0, 6·6	–	5·42	5·0, 5·8	–	0·24
Lipids (g/kg per d)	1·95	1·8, 2·1	2·22	2·1, 2·3	0·017	2·05	1·9, 2·2	0·63	0·38
SFA (g/kg per d)	0·61	0·54, 0·68	0·69	0·66, 0·73	0·04	0·63	0·57, 0·68	0·91	0·66
MUFA (g/kg per d)	0·77	0·69, 0·86	0·90	0·85, 0·95	0·011	0·89	0·82, 0·97	0·058	0·027
PUFA (g/kg per d)	0·47	0·41, 0·53	0·53	0·50, 0·57	–	0·45	0·40, 0·5	–	0·63
Cholesterol (mg/d)	542	476, 608	605	567, 643	–	524	476, 572	–	0·76
Trans fats (g/d)	1·83	1·6, 2·1	2·14	2·0, 2·3	0·033	2·03	1·8, 2·3	0·36	0·20
Fibre (g/d)	30·6	28·4, 32·8	32·8	31·5, 34	–	30·2	28·3, 32·1	–	0·90
Na (mg/d)	4217	3920, 4515	4816	4616, 5015	0·002	4102	3821, 4384	0·82	0·74

The *P*-value for the univariate comparison against the lowest educational level (Dunnett’s test) is shown only when global ANOVA was significant.

**Fig. 1:**
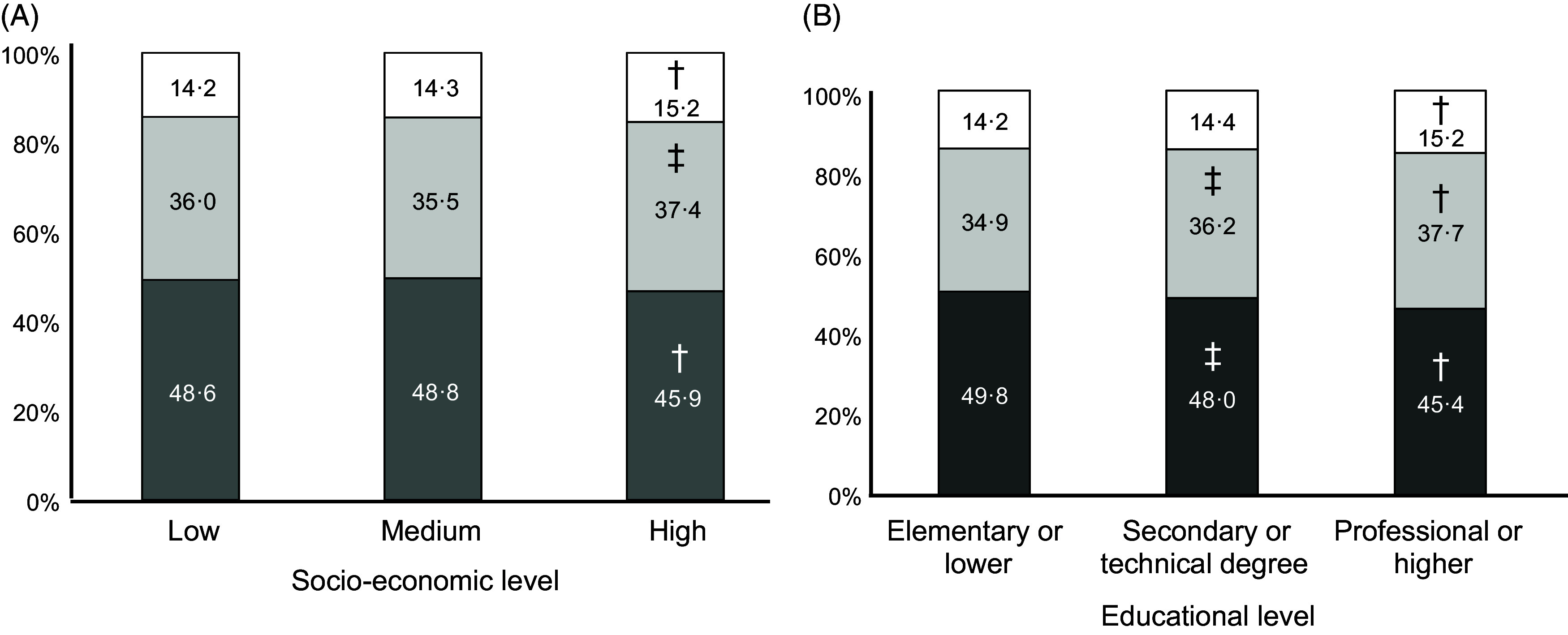
Estimated intake of macronutrients by socio-economic stratum (Panel A) and educational level (Panel B) (*n* 1491 for Panel B). Each coloured area represents the proportion of total energy intake from the corresponding macronutrient. ^†^ *P* < 0·001 *v* lowest category, ^‡^ *P* < 0·01 *v* lowest category. 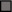, carbohydrates; 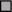, lipids; 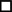, protein

**Fig. 2:**
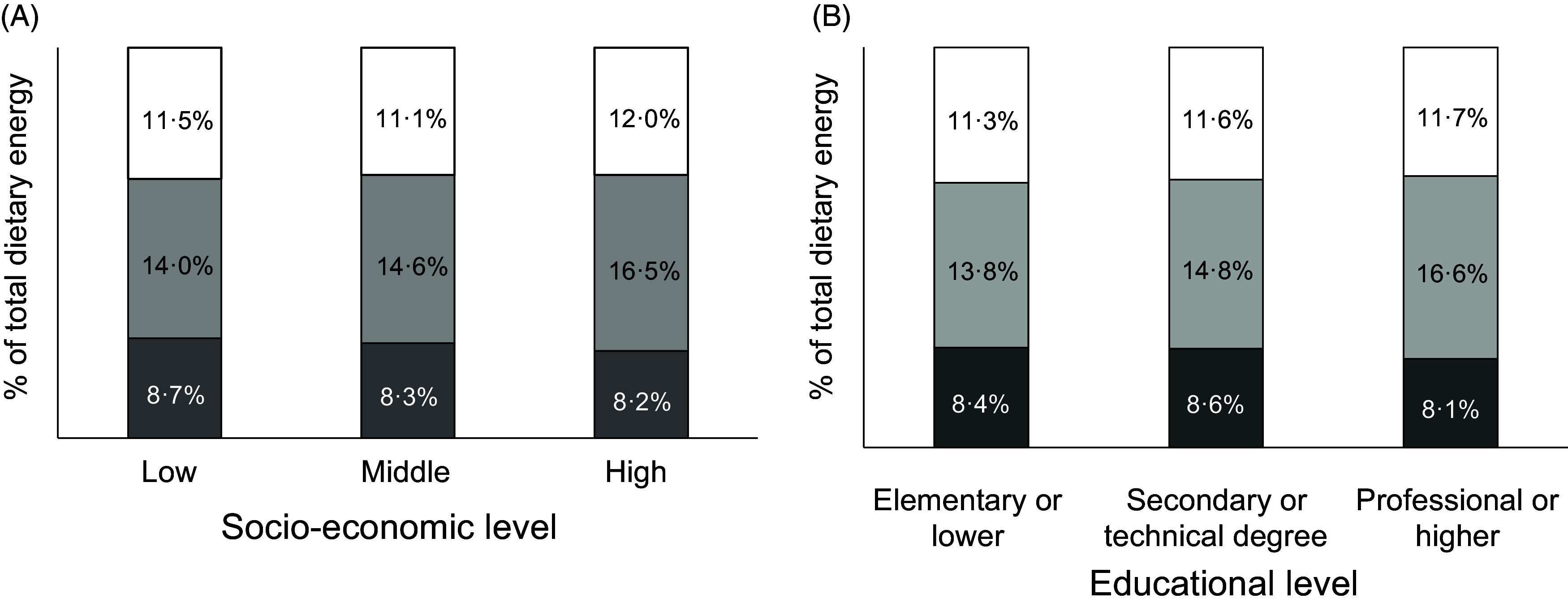
Estimated contribution of different dietary fat types to total energy intake by socio-economic stratum (Panel A) and educational level (Panel B) (*n* 1491 for Panel B). SFA, saturated fats; MUFA, monounsaturated fats; PUFA, polyunsaturated fats. ^†^ *P* < 0·001 *v* lowest category, ^‡^ *P* < 0·01 *v* lowest category. 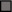, PUFA; 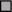, MUFA; 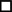, SFA

As expected, the intake of all macronutrients per kg body weight decreased markedly across age groups: 73 % for carbohydrates, 69 % for total lipids and 70 % for protein comparing age group 60–75 to age group 2–11. The macronutrient composition of the diet changed only slightly with SES, with significantly less energy coming from carbohydrates (45·9 *v* 48·6 %, *P*-trend <0·001) and more from lipids (37·4 *v* 36·0 %, *P*-trend = 0·031) and protein (15·2 *v* 14·2 %, *P*-trend <0·001) in the highest compared with lowest SES (Fig. 1, Panel A). The trend towards less carbohydrate intake was somewhat more pronounced, and also significant, across educational levels: energy from carbohydrates went from 49·5 % in the lowest to 45·4 % in the highest category (*P*-trend <0·001, Fig. 1, Panel C). The proportion of MUFA increased with higher SES, at the expense of SFA and PUFA (*P*-trend < 0·001, Fig. 2, Panel A). The trend towards more energy coming from MUFA and less from PUFA was also significant as educational level increased (*P*-trend < 0·001, Fig. 2, Panel B).

**Fig. 3:**
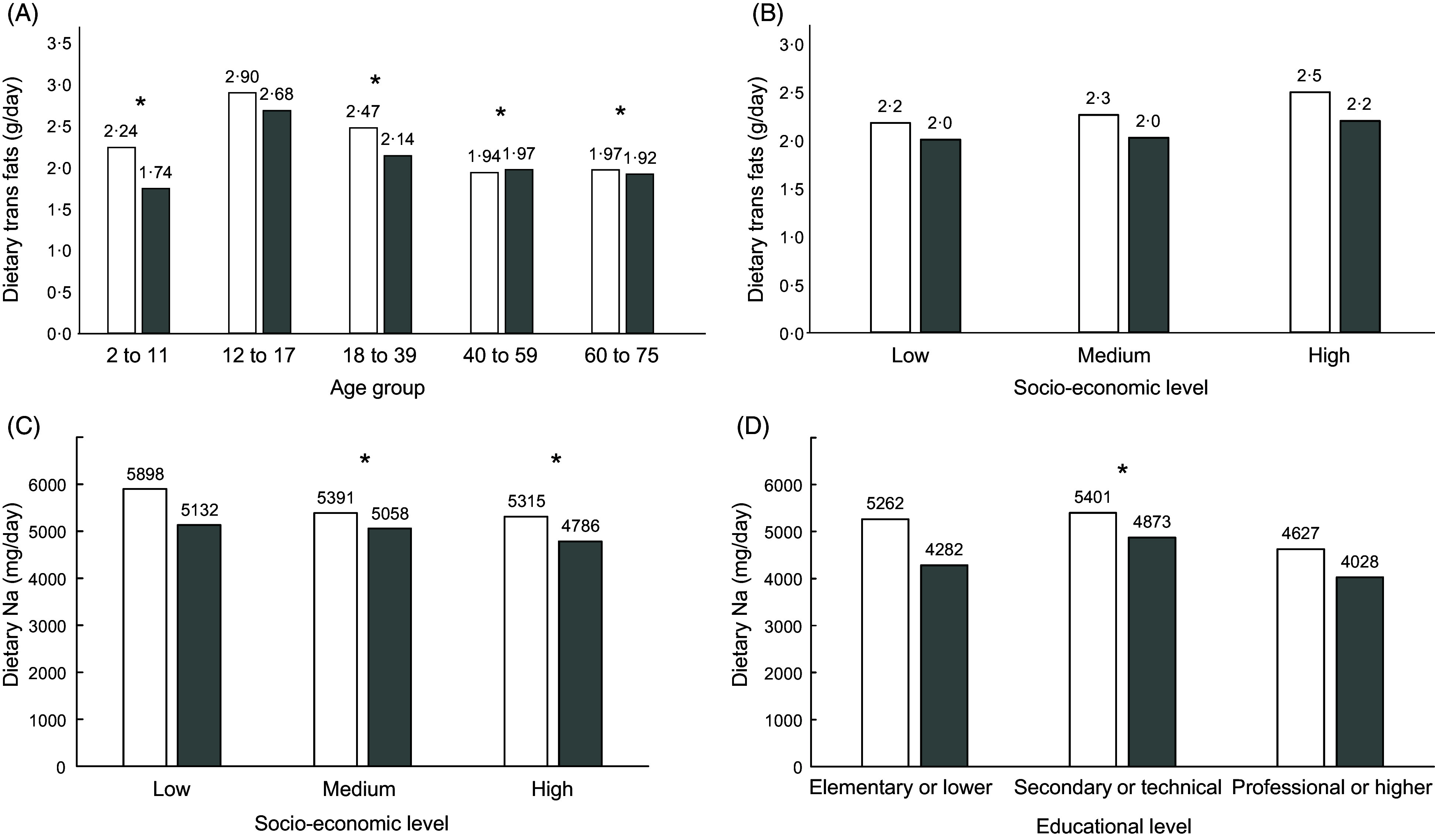
Estimated dietary intake of trans fats by age group (Panel A) and socio-economic stratum (Panel B), and dietary intake of Na by socio-economic stratum (Panel C), and educational level (Panel D) (*n* 1491 for Panel D). In all panels, significant *P*-values for the comparison *v* the reference category are indicated by an asterisk. This test involves all participants (male and female) in each category. In Panel A, the reference category is age 12–17, in Panels B–D, the reference category is the lowest. 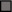, male; 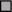, female

The intake of trans fats was particularly high in adolescents relative to other age groups (Fig. 3, Panel A). The intake of trans fats increased with SES only among men. This linear trend did not reach statistical significance (*P*-trend = 0·19), but the pairwise comparison between extreme SES categories did (*P* = 0·04). Trans fat intake was also higher among more educated men, going from 1·86 g/kg per d in the lowest to 2·26 g/kg per d in the highest education category (*P*-trend = 0·023). (Fig. 3, Panel B). Na intake was negatively correlated with SES, going from 5262 mg/d in low SES to 4627 mg/d in high SES among males (*P*-trend = 0·005), and from 4282 mg/d to 4028 mg/d among females (*P*-trend < 0·001); the same significant trend was observed for Na intake across educational levels in both sexes (Fig. 3, Panels C and D). The absolute difference in Na intake between males and females became smaller with higher education.

## Discussion

In this population-based study of the main Colombian cities, we assessed the influence of SES and educational level on the nutritional attributes of the diet. Our results show that relative to body size, the diet of individuals in a low SES is characterised by a higher intake of Na, carbohydrates and total energy. Education also had an impact on diet composition, so that more educated men consumed slightly less carbohydrates and more detrimental (trans) fats, while more educated women consumed more of the beneficial MUFA. Such information is a valuable input for health policy makers not just from Colombia but from other countries with similar demographics and economic development.

The last two decades have witnessed an unprecedented increase in prosperity in Colombia, a change that should manifest itself in the educational attainment of the population, if social mobility is to be achieved and social inequality mitigated. Despite recent increases in participation rates, the educational system remains heterogeneous in quality, and there is a narrow bottleneck in access to tertiary education^(28)^. According to OECD data, only 25 % of the poorest Colombians went to university in 2016, while 61 % of the richest Colombians did^(29)^. Data collected between 1998 and 2007 showed that Colombian adults with only primary education had more than twice the mortality rates of those with post-secondary education. Further, there is a trend towards reduced mortality over time in the whole population, but the declines are larger for higher-educated men and women^(30)^.

We used two measures of SEP, one at the household level (SES) and other at the individual level (educational level). According to the taxonomy proposed by Howe *et al.*^(31)^, SES may be considered an asset-based measure, as the classification of household is based on the availability of utilities like electricity, sewage and running water, access routes, topography, land valuation and property characteristics. These elements may be considered a better indicator of social position in low- and middle-income countries, in which income or consumption data may be volatile and unreliable^(31)^. On the other hand, education provided us with a different aspect of SEP, one concerned with the achievement of general literacy, which tends to be a very good correlate of health literacy. The fact that we registered the highest educational milestone achieved helped prevent the confounding effect of individuals who take longer to complete educational cycles^(31)^. The association of different indicators of SEP with dietary behaviour may vary greatly across populations, especially when comparing low- and middle-income countries with more affluent societies. For example, a very large study in France found that a lower income was associated with larger intakes of meat and poultry^(32)^, both sources of dietary protein, while in our results a higher consumption of dietary protein was observed for females with higher SES.

Even though education and income tend to be correlated, in our study of urban residents SES was associated with more aspects of dietary intake than educational level, particularly energy, carbohydrates and Na intake. Meanwhile, educational level was a more important correlate of MUFA and trans fat intake. This has not necessarily been the case in nation-wide studies from other countries like Brazil^(33)^ or some European nations^(34,35)^, albeit methodological differences preclude direct comparisons. Indeed, compared with income and occupation, education has a greater effect on health-related behaviours like smoking and physical activity^(5)^, an effect that has been dubbed ‘education gradient’^(36)^. In a study in five Latin American countries, the factors wealth, ability to act and cognition explained between 50 and 70 % of this education gradient in dietary behaviour^(37)^. Evidence from China suggests that, in certain social contexts, even the educational level of the spouse may have a relevant influence on dietary behaviours, especially among women^(38)^. In a study undertaken in five Italian cities, being in the highest tertile of educational level was associated with several relevant qualitative dietary behaviours, among them a lower intake of complex carbohydrates and sugary drinks and a higher intake of dairy products, fish, fruits and vegetables^(39)^. Interestingly, a recent cross-sectional study in Portugal found a higher educational level to be the main correlate of good adherence to a Mediterranean dietary pattern^(40)^. In the Polish National Multi-Centre Health Examination Survey, a composite ordinal score that combined educational level and household income was correlated positively with a better overall diet quality^(41)^. These associations may translate into later impact on harder outcomes. In a long-term follow-up of a population study in Norway, a large difference in total mortality rates was found between the lowest and highest educational level. Health-related behaviours, of which dietary habits constituted a central part, explained between 38 and 45 % of this difference^(42)^. A longitudinal analysis of nationally representative data from the Australian National Nutrition and Physical Activity Survey between 1995 and 2013 showed that individuals in a better SEP (assessed by educational level, household income and area-level disadvantage) tended to improve aspects of their dietary behaviour over the study follow-up, including intakes of energy, total fat, saturated fat and fruits^(43)^. These results suggest that in some countries the impact of social inequalities on dietary intakes may be getting more pronounced over time. The impact of education on self-care attitudes, habits and eventually health may be enhanced in Latin America, as access to higher/tertiary education is quite limited^(44,45)^.

Educational level seemed to have a greater impact on the quality of dietary fats. Although sex differences in dietary intake are well known^(46)^, differences in the education–diet association between sexes have been less studied^(8,10,34,47)^. Given that higher education reduces gender inequality in self-rated health and mortality^(48)^, our results support education as a nutrition public policy^(49)^ and as an instrument for social progress^(50)^.

A lower income has been associated with greater intake of energy-dense foods, especially those rich in carbohydrates^(51,52)^. A study in US children found that drinking sugary beverages was negatively associated with both SES and work stability of the parents^(53)^. A huge body of evidence supports the negative influence of a high carbohydrate intake on cardiovascular and metabolic health^(54,55)^, labelling it as one of the main sources of the current obesity epidemic^(56,57)^. Excess carbohydrate ingestion may be related to easy access to pastries and other sugary foods and drinks, plus limited access to protein-rich foods and sugar substitutes^(58)^.

An interesting result was that a better SEP was associated with both a greater intake of MUFA (present in a Mediterranean dietary pattern)^(59)^ among women and a greater consumption of trans fatty acids (commonly present in ultra-processed and fast foods)^(60,61)^. Even though this pattern seems contradictory, several other studies have found the same association^(62,63)^. The larger MUFA intake in more educated individuals in Latin American countries could be explained by the influence of numerous public health campaigns about the negative effects of SFA in cardiovascular and neurological health, combined with the higher cost of MUFA-rich oils^(62,64)^. Concerning trans fats, the influence of US culture and habits about practical and cheap foods may be greater in higher income groups^(63)^.

As in our investigation, many other studies have reported higher consumption of trans fats among younger persons^(62,63,65)^. The intake of trans fats has been increasing rapidly during the last years^(62,65)^, despite their well-known harmful effects on CVD and mortality^(66)^. Different factors may explain this phenomenon in underage individuals: first, there is a positive correlation between ingestion of trans fats or sugar-sweetened beverages and time spent watching television^(67)^. Second, there has been a rise in fast food advertising, and third, such publicity appears mostly in time slots and channels addressing children, adolescents and young adults^(68–70)^. Thus, the regulation of advertising to young audiences in terms of quantity and content is a priority target for public health policy.

The conflicting results from different countries concerning the association between SES and dietary intake can reflect the ‘nutrition transition’ concept. Our results illustrate that in cities from middle-income countries like Colombia, low SES groups tend to have a higher energy intake because of increased access to energy-dense foods. Meanwhile, high SES groups tend to avoid such foods because of: (i) greater exposure to information about the increasing rates of obesity and its consequences^(71,72)^; (ii) ability to afford non-energy-dense foods like fruits and vegetables, which may be expensive in an urban environment^(73)^; (iii) an interest in projecting a socially desirable image of a healthy lifestyle and self-care and^(74)^ and (iv) the influence of body image models from the mass media that portray leanness as a sign of success and self-fulfilment^(75)^. In this sense, findings from China (1989–1997) are similar to ours^(8)^. By contrast, countries with different results^(8–10,76)^ could be experiencing an earlier phase of the nutrition transition, in which income is the main determinant of access to nourishment, so low SES groups are exposed to food scarcity. Also, our findings may not reflect the situation in rural areas of Colombia or of other countries at a similar stage of the nutrition transition.

The main strengths of our study include the probabilistic, population-based sample, the representation of geographically and culturally different regions of the country and the use of a FFQ that was adapted to Colombian foods and preparations. The collection of information in a supervised manner by qualified staff and the use of several quality checks helped improve the reliability of study data. The main limitations of the study are those inherent to its cross-sectional design, including the impossibility to explore the long-term health effects of the observed differences in dietary behaviour by SEP. Additionally, our results do not represent dietary habits in rural areas of Colombia, and we did not take into account foods consumed by less than 30 % of the Colombian population. We employed two different indicators of SEP, one of them focused on assets (SES) and the other on education. Our results do suggest that even though these indicators may be correlated, their association with at least some aspects of dietary intake is different. SES has the particularity of being defined by household and not by individual, so within-household variations in income or assets among respondents were not captured by this measure. Nonetheless, most of the variation in income and assets in Colombia (and presumably in other countries) is explained by between-household differences. In a population-based survey in Alberta, Canada, household income was a much better predictor of future survival and health status than respondent income^(77)^. Self-reported educational level may be subject to misreporting, usually of a higher level, a problem that could weaken associations between the reported variable and dietary intake outcomes. However, ascertaining the maximal educational level of all participants would not be logistically feasible, as there is not a central registry of all degrees granted by Colombian institutions, so this was the best approximation possible under realistic circumstances.

In summary, our results show the strong influence that SEP has on usual diet in the main Colombian cities. Individuals living in lower SES consume significantly more total energy, more carbohydrates and more Na, characteristics extensively documented to correlate with greater risks for obesity, diabetes, CVD and total mortality. Also, this dietary pattern is very likely to be fuelling the current rise of overweight and obesity among the economically disadvantaged in Colombia^(15)^. A higher educational level was associated with greater intake of cardioprotective MUFA among women but also of the very harmful trans fats among men. Thus, health policies for the lower SES segments in our local context should focus more strongly on strategies aimed at the energetic adequacy of the diet, while those aimed at more educated segments should have a stronger emphasis on the quality of dietary fats.
